# Analysis of cerebrovascular dysfunction caused by chronic social defeat in mice

**DOI:** 10.1016/j.bbi.2020.05.030

**Published:** 2020-05-12

**Authors:** Michael L. Lehmann, Chelsie N. Poffenberger, Abdel G. Elkahloun, Miles Herkenham

**Affiliations:** a Section on Functional Neuroanatomy, Intramural Research Program, National Institute of Mental Health, National Institutes of Health, Bethesda, MD 20892, USA; b Division of Intramural Research Programs Microarray Core Facility, National Institutes of Health, Bethesda, MD 20892, USA

**Keywords:** Inflammation, Neuroimmune, Blood-brain barrier, Endothelia, Cerebrovasculature, Leukocyte, Angiogenesis, Fibrinogen, Psychosocial stress, Depression

## Abstract

Psychological stress and affective disorders are clinically associated with hypertension and vascular disease, but the biological links between the conditions have not been fully explored. To examine this relationship, we used chronic social defeat (CSD) stress, which produces anxiety-like and depressive-like behavioral declines in susceptible mice. In such mice, CSD also produces cerebrovascular microbleeds in scattered locations. Here, we showed further evidence of vascular pathology and blood–brain barrier breakdown by visualizing plasma immunoglobulins and erythrocytes within the parenchyma and perivascular spaces of CSD brains. To further characterize the impact of stress on the cerebrovasculature, brain endothelial cells (bECs) were isolated, and global gene expression profiles were generated. Bioinformatic analysis of CSD-induced transcriptional changes in bECs showed enrichment in pathways that delineate the vascular response to injury. These pathways followed a temporal sequence of inflammation, oxidative stress, growth factor signaling, and wound healing (i.e., platelet aggregation, hemostasis, fibrinogen deposition, and angiogenesis). Immunohistochemical staining for markers of fibrinogen deposition and angiogenesis confirmed the existence of the markers at the sites of vascular disruptions. Recovery after CSD cessation was marked by recruitment of leukocytes perhaps participating in vascular repair. The data suggest that co-morbidity of affective disorders and vascular diseases may be attributed in part to a common link in altered endothelial cell function.

## 1. Introduction

Major depressive disorder and related psychopathologies are a leading cause of disability in the world (GDB, 2018). These disorders have a complex multifactorial etiology that includes social risk factors such as psychosocial stress (Kendler and Gardner, 2010; Keyes et al., 2011; Rice et al., 2017). The association between stress and depression can be studied in rodents using validated behavioral paradigms such as chronic social defeat (CSD) (Golden et al., 2011). These models permit study of brain circuits, responsive cell types, and molecular pathways that determine the effects of stress on behavior. Analysis of structural changes precipitated by chronic stress may point to potential therapeutic targets for treating mental disorders.

Research using CSD shows a surprising involvement of non-neuronal cells responding to chronic defeat (Bonnefil et al., 2019; Lehmann et al., 2017; Lehmann et al., 2019; Stein et al., 2017). Microglial cells, the major immune cell type, have been the focus of much attention. We recently showed that mice that are susceptible to chronic defeat (CSD-S)—that display increased anxiety and social avoidance, as opposed to mice resistant to defeat effects (CSD-R)—have increased microglial gene expression indicative of inflammation, oxidative stress, extracellular matrix remodeling, and phagocytic activity (Lehmann et al., 2018). The expression patterns suggested that CSD-S microglia respond to and/or drive blood–brain barrier (BBB) breakdown. To investigate this possibility, markers of BBB integrity were employed. Indeed, histological analyses showed that CSD caused local breaks in BBB integrity manifested as microhemorrhages, or microbleeds, selectively in susceptible mice (Lehmann et al., 2018; Menard et al., 2017). These findings support clinical research showing that stress is a risk factor for cardiovascular (Everson-Rose et al., 2014; Rosengren et al., 2004; Steptoe and Kivimaki, 2012) and cerebrovascular disease (Burrage et al., 2018).

Many factors might contribute to breakdown of BBB at the level of small cerebral vessels, including peripheral and/or central inflammation, oxidative stress, extracellular matrix degradation, and elevated blood pressure (Low et al., 2019; Ungvari et al., 2017). Such factors are prevalent in hypertension, small vessel disease, and atherosclerosis (Iadecola and Gottesman, 2019), and some are associated with psychological stress disorders (Marsland et al., 2017; Schiavone et al., 2013) and depression (Black et al., 2015; Greaney et al., 2019; Kohler et al., 2017; Salim, 2014). Endothelial cells, which line the blood vessel lumen, are the principal component of the BBB (Blanchette and Daneman, 2015). They are the main regulators of vascular homeostasis (Michiels, 2003), and they respond vigorously to injury (Eelen et al., 2015). They are thus major contributors to the production and resolution of stress-induced microbleeds in defeated animals. We undertook a transcriptomic investigation of brain endothelial cells (bECs) at multiple time points during CSD and after termination of CSD to track gene expression patterns, followed by histochemical confirmation of the significant identified events occurring in the vasculature.

## 2. Materials and methods

### 2.1. Animals

All procedures were approved by the National Institute of Mental Health Institutional Animal Care and Use Committee and conducted in accordance with the National Institutes of Health guidelines. Behavioral experiments were performed using male CD-1 retired breeder mice and 8–10 week-old male C57BL/6N mice (Charles River Laboratories). All test animals were group-housed in pathogen-free conditions in a 12-h light/dark cycle with lights off at 9:00 AM. Food and water were provided *ad libitum.* Behavioral testing was done in the dark phase under dim red lighting at approximately 40 lx.

### 2.2. Chronic social defeat (CSD)

CSD was used to model the effects of chronic psychosocial stress in mice. As in our previous studies (Lehmann et al., 2013a; Lehmann et al., 2018), an experimental C57BL/6N mouse was housed for 1, 7, or 14 days in the home cage of an aggressive, territorial male CD-1 mouse with a perforated transparent acrylic partition separating the mice. Mice were randomly assigned to each treatment group. The partition was removed for 5 min each day to allow agonistic encounters between the mice. Home cage (HC) mice were housed 2 per cage with a perforated partition permanently separating the mice. In the recovery group (CSDrec), mice were placed back in HC condition for 7 days, 2 per cage with a perforated partition permanently separating the mice, and cagemates were randomly assigned.

### 2.3. Behavioral analysis

All mice except groups receiving a single defeat were phenotyped on the day prior to experimental endpoints to map behavioral responses to stress conditions. Behavioral testing was conducted 2 h after the last defeat session for mice in CSD housing. The two tests were performed 1 h apart.

#### 2.3.1. Social interaction (SI) test.

All behavioral tests were performed as described previously (Lehmann et al., 2013a; Lehmann et al., 2018). To determine the SI quotient, the mouse was placed for 15 min in a 50 cm × 50 cm × 50 cm arena containing two perforated acrylic cylinders. One cylinder contained a CD-1 mouse and the other was empty. The position of the mouse was recorded from above and subsequently automatically analyzed with TopScan (CleverSystems, Inc., Leesburg, VA). The SI quotient was determined by dividing the time spent investigating the cylinder containing the CD-1 mouse by the time spent investigating the empty cylinder. Lower scores were indicative of asocial behavior.

#### 2.3.2 Light:dark (LD) box test.

The LD test was conducted using an acrylic box (50 cm × 25 cm with 30 cm walls) consisting of a dark (one-third of the box) and a lit (~40 lx) compartment (two-thirds of the box). An open door divided the compartments. Each mouse was placed in the light compartment and allowed to freely move within the compartments for 10 min. The time spent in the light compartment and number of crosses between the light and dark sides were scored (TopScan, CleverSystems, Inc.). Low scores were indices of anxiety-like behavior.

### 2.4. Brain endothelial cell (bEC) isolation

Sixteen h after the last defeat session, whole brain minus cerebellum, brainstem, and meninges were dissected from phenotyped HC and CSD mice perfused with 0.9% saline. CSD mice were selected that had SI and LD scores indicative of stress-susceptibility (Lehmann et al., 2018). Minced brain was added to components of the Neural Tissue Dissociation Kit (Miltenyi Biotec), including 2850 μl of Buffer X and 75 μl of Enzyme P and further minced through gentle trituration with a 1000 μl pipet tip. After 17 min incubation at 37 °C with slow rotation, 30 μl of Buffer Y and 15 μl Enzyme A were added; the mixture was triturated 10x, gently to prevent bubbles, with a 1000 μl pipet, rotated slowly for 12 min at 37 °C, passed 10 times through a 20-ga needle, and incubated for a final 10 min at 37 °C. After this step, samples were kept ice cold for the remainder of the isolation. 20 ml of cold PBS (plus Mg^+^ and Ca^++^) was added to the mixture, passed through a 70 μm filter, and pelleted at 300 g for 5 min. Myelin was removed following Miltenyi protocols (Miltneyi Biotec). The resulting cell suspension was blocked with CD16/32 (BioLegend cat #101302) and goat serum (Sigma cat #G9023) (5% each in 200 μl HBSS/0.1%BSA) for 10 min on ice, labeled with CD31 PE/Dazzle (BioLegend cat #102430) for 25 min on ice, washed, pelleted, resuspended in 1 ml HBSS/0.1% BSA and further labeled with DAPI (dead exclusion) (Thermo cat #62248) and DRAQ5 (DNA inclusion) (Thermo cat #62251). CD31^+^DRAQ5^+^DAPI^−^ single cells were isolated and captured with a FACS Aria sorter (BD Biosciences) into Trizol LS (Life Technologies cat #10296028) and stored at −80 °C for RNA isolation. Total time from perfusion to FACS collection was approx. 150 min and yielded 2 × 10^5^ viable single cells. Samples from multiple conditions were harvested on each collection day. The following number of samples were sorted and collected from each condition: HC, 9; CSD1, 5; CSD7, 5; CSD14, 8; CSDrec, 9.

### 2.5. Microarray

Samples were prepared according to Affymetrix protocols (Affymetrix, Inc.) and as described previously (Lehmann et al., 2018; Lehmann et al., 2017). RNA quality [RNA integrity number (RIN) > 9] and quantity (> 300 ng) were ensured using the Bioanalyzer (Agilent, Inc.) and NanoDrop (Thermo), respectively. Samples that fit these two criteria were considered for the microarray and then randomly selected in cases where multiple samples met criteria. 300 ng of total RNA was prepared according to Affymetrix protocols and hybridized to Affymetrix Clariom D mouse chips (cat #902513). The chips were washed and stained by the Affymetrix Fluidics Station using the standard format and protocols. Probe-level data for 29,215 mouse gene fragments per hybridized cDNA were generated using an Affymetrix Gene Chip Scanner 3000. The resulting .cel files were imported into Partek Genomics Suite version 6.6 (https://www.partek.com/partek-genomics-suite/), and Robust Multichips Analysis (RMA; part of Partek Genomics Suite software, version 6.6 beta) background correction and quantile normalization were performed. A Median Polish was done for probeset summarization, and the data were Log2 transformed. Data quality was assessed by visual inspection with a Tukey box plot, covariance-based Principal Component Analysis (PCA), scatter plot, and correlation-based Heat Map.

### 2.6. Bioinformatics analysis

#### 2.6.1. Partek.

Normalized data sets were compared in Partek by ANOVA with false discovery rate (FDR) correction (q < 0.05) to assess differential transcript expression across behavioral phenotypes. Data were visualized by heatmap with transcripts and samples organized by hierarchical clustering on log transformed and z-score transformed data. Both samples and genes were hierarchically clustered based on the Euclidean and Average Linkage method. Heatmaps (samples on rows and genes on columns) were drawn from the different clusters identified. The colors were assigned based on relative standardized expression values (mean of 0, standard deviation of 1; performed on the fly), with blue indicating low and red indicating high levels of a variable (i.e., low or high expression levels).

#### 2.6.2. Cytoscape.

Differentially expressed (DE) transcripts were revealed for each treatment condition (HC vs. CSD1, CSD7, CSD14, CSDrec) and transcripts with fold change ≥ 1.3 × than HC were imported into ClueGo (version 2.5.4) (Bindea et al., 2009) using the Cytoscape environment (3.7.1) (Shannon et al., 2003). Multiple marker lists were used for this analysis, each treatment was imported into a single list. DE transcripts were used to generate biological processes networks using gene ontology (GO) annotation from the European Bioinformatics Institute (https://www.ebi.ac.uk/GOA). The GO interval was between 3 (Min level) and 9 (Max level). The Kappa score was 0.5. For the enrichment of biological terms and groups, we used the two-sided (Enrichment/Depletion) tests based on the hyper-geometric distribution. We set the statistical significance to 0.05 for transcriptomic result, and we used the Bonferroni adjustment to correct the p-value for the terms and the groups created by ClueGO. The leading group term is based on highest significance vs. cluster. ClueGO represents the results as a network of GO terms connected with edges that reflects the relationships between the terms based on the similarity of their associated genes. This allows tools from graph theory to be used to reorganize the layout of the network to uncover communities inside GO term networks, e.g., metabolic processes or angiogenesis. Color gradients were used to reflect dominance of that node by a particular treatment, determined by the gene proportion of each treatment condition associated with the term. The percent of differentially expressed (DE) transcripts in select GO terms was calculated for each treatment condition and expressed as dominance.

Dominance(group,term)=((#genes(group,term))÷#genes(unique,term))×(1−log(#genes(total,term)÷#genes(unique,term))÷log(#genes(total,term)))×100

Representative GO terms for each community were manually curated, and Fishers Exact test was run to calculate Dominance and GO term pValue for each treatment condition to reveal changes in function over time.

#### 2.6.3. Ingenuity Pathways Analysis (IPA).

IPA was used for revealing top upstream regulators for each list of differentially expressed transcripts. Of several tools available for mining array data, IPA uses a high percentage of curation by experts for multiple sources of information (see https://www.qiagenbioinformatics.com/products/ingenuity-pathway-analysis/).

#### 2.6.4. Gene Set Enrichment Analysis (GSEA).

Published microarray datasets were mined to reveal phenotypes in the present dataset using GSEA (http://broadinstitute.org/GSEA) according to methods previously described (Subramanian et al., 2005). The GSEA algorithm computes a ranked list of all genes from a microarray comparison between two conditions and identifies whether individual members of an *a priori* functionally defined gene set are enriched or randomly distributed across the whole ranked gene list, using a modified Kolmogorov-Smirnov statistic and generating an enrichment score. FDR *q*-value was set at < 0.05, and statistical significance adjusted for multiple hypothesis testing was *p* < 0.05. A gene set-based permutation test of 1000 permutations was applied, and genes were ranked according to Student’s *t* statistic. All other parameters were set to GSEA defaults.

### 2.7. Immunohistochemistry

Sixteen h after the last defeat and 5 min prior to harvest, isoflurane-anesthetized mice were intravenously (*retro*-orbitally) injected with 0.1 ml DyLight 594-labeled Tomato Lectin (Vector Labs). Deeply anesthetized mice were perfused with 20 ml, 0.9% saline followed by 15 ml ice-cold 4% paraformaldehyde. Brains were removed, post-fixed overnight, followed by 25% sucrose in phosphate-buffered saline (PBS) for 24 h. Coronal brain slices (30-μm thick) were collected on a freezing microtome. Six equally distributed sections from + 1.94 to −3.40 mm relative to bregma were examined for each stain from each animal. Several protocols were utilized, and all used the following regimen: free-floating sections were washed 3x for 10 min each, blocked for 1 h at RT, incubated overnight at RT with the primary antibody at 1:1000 dilution, washed, incubated for 2 h with secondary antibody at 1:500 dilution, washed, mounted, and coverslipped with PVA-DABCO (Sigma).

For erythrocyte stains, sections were washed with 0.1% Triton X-100 in 0.1 M PBS, blocked in 4% normal goat sera (NGS), incubated overnight with rat-anti Ter119 (erythrocyte marker, Abcam cat #ab91113) followed by AlexaFluor 488 goat anti-rat IgG secondary antibody (Life Technologies cat #A11006).

In studies examining immunoglobulin G (IgG), sections were blocked in 4% NGS/1% BSA in 0.4% TX-PBS for 1 h, incubated 1:500 in AlexaFluor 488 Goat anti Mouse IgG (Life Technologies cat #A11001).

Studies examining fibrinogen and angiogenesis, sections were treated as above and then incubated with a fibrinogen antibody (rabbit anti-human fibrinogen, DAKO cat #A0080) and a combined cocktail of angiogenesis markers (CD202 cat# 124001, CD105 cat# 120401, and VEGFR2 cat #136401, all rat anti-mouse, BioLegend). Sections were washed with 0.1% Triton X-100 PBS, blocked with 5% BSA in 0.3% Tx-100/PBS, incubated overnight in 1% BSA/0.1% Triton X-100/PBS with primary antibodies, then incubated with AlexaFluor 633 goat antirabbit IgG (Life Technologies cat #A21071) and AlexaFluor 488 goat anti-rat IgG (Life Technologies cat #A11006) secondary antibodies.

### 2.8. Confocal microscope analysis

Each coronal slice of immunofluorescent stained tissue was captured in its entirety with stitching on a Zeiss 780 confocal microscope with a 10x apochromat objective. Higher quality images were captured with 20x and 40x oil objectives. To map the number and location of vascular disruptions marked by Ter119 and IgG staining in HC and CSD mice, 10x whole-slice scans were manually annotated in Zen Blue (Zeiss) by two different observers blinded to all conditions. The annotated images with mapped vascular changes were imported into Adobe Illustrator and plotted onto hand-drawn brain maps. In examination of vascular responses to CSD marked by fibrinogen or angiogenesis stains, whole-slice images were analyzed using Volocity 6.3 (PerkinElmer). The areas occupied by vascular labeling with fluorescent tomato lectin and stains of interest were subjected separately to thresholding in their respective channels. First, a threshold two standard deviations higher than the median value was applied to immunostains of interest, then the area of stain was normalized to the area of tomato lectin labeling. The % fibrinogenic + or % angiogenic + vessels were determined by dividing the area of stain by the area of tomato lectin-labeled vessels.

### 2.9. Statistics

Data were summarized as mean ± SEM, and differences among experimental conditions were considered statistically significant when the p value was ≤ 0.05. GraphPad Prism Version 7 and MS Excel Version 16 was used to analyze data using Student's two-tailed *t* tests, one-way ANOVA, simple linear regression, Pearson’s correlation coefficient test, or Fishers Exact test where appropriate to assess between-subject comparisons, for example, behavioral, transcriptional, or biological assay score. Statistical analysis for Microarray and GSEA is described above

## 3. Results

### 3.1. Behavior

**Fig. 1.**
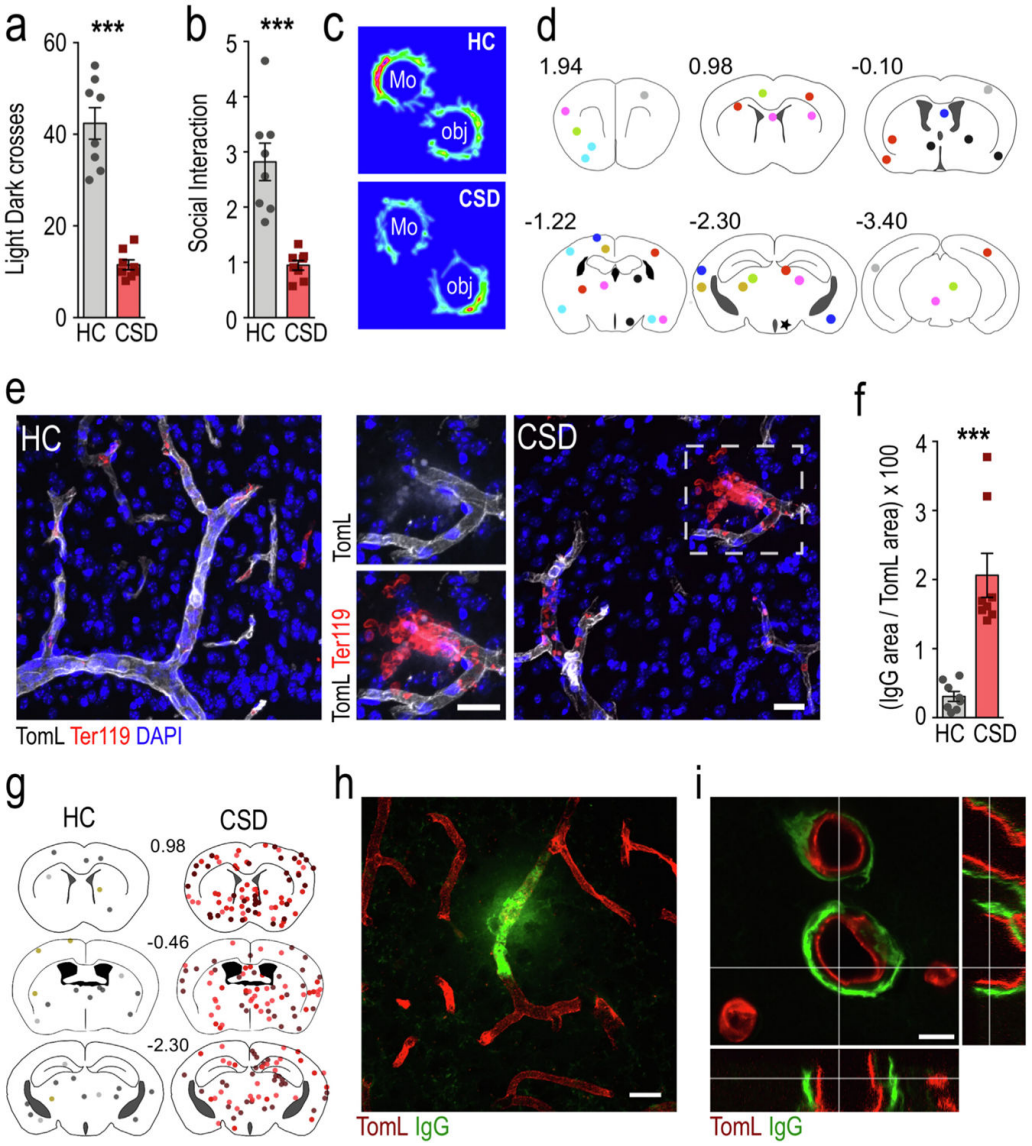
Chronic social stress causes substantial elevations in microbleeds and perivascular IgG deposition in brain. a,b, CSD-exposed mice show significant declines in crosses between chambers in the light:dark (LD) test (a), and significant reductions in social exploration in the social interaction (SI) test (b). c, representative heat maps of behavior for CSD and HC mice in the SI task. d, anatomical locations of microbleeds indicated by extravascular erythrocyte clusters. The dots are color-coded for individual mice in the CSD group (n = 8), a single bleed was detected in a HC brain (marked with a star). e, examples of tomato lectin (TomL)-stained vasculature in HC and CSD brains and isolated bleeds in CSD brain; inset shows extravascular erythrocyte cluster. f, perivascular IgG deposits are substantially elevated in CSD brain (n = 8). g, IgG deposits occur in anatomically diverse regions (dots are color-coded for 3 mice in each group to demonstrate effect). h,i, Confocal images showing IgG leaks (h) outside of tomato lectin-stained vasculature and perivascular deposition (i) in CSD brain. N = 8 per group unless where noted. *** p < 0.0001. Abbreviations: mo, mouse; obj, object. Scale bars = 20 μm for e,h and 10 μm for i.

Our previous work in chronically defeated animals suggested that CSD causes BBB dysfunction in a subset of mice susceptible to CSD (CSD-S) (Lehmann et al., 2018). In the current study we mapped the leakage of erythrocytes and IgG into perivascular spaces and parenchyma in CSD mice (Fig. 1) to further characterize the extent of BBB dysfunction and to determine if CSD causes cerebral microbleeds. The robust emergence of anxiety-like and asocial behaviors was observed in defeated mice, manifested by significant declines in both light:dark (LD) crossings (Fig. 1a) (t = 8.56, p = 0.0001) and social interaction (SI) ratios (Fig. 1b,c) (t = 5.37, p = 0.0001) compared to unstressed control mice (HC). These are thus categorized as CSD-S mice by the previous terminology (Lehmann et al., 2018).

### 3.2. Histology of microbleeds

Coupled with changes in behavior, significant increases in extravascular erythrocyte clusters (microbleeds) marked by Ter119 antibody were observed in CSD (0.77 ± 0.1 microbleeds/slice) versus HC brain (0.02 ± 0.02 microbleeds per slice) (t = 7.29, p = 0.0006). Microbleeds were distributed throughout brain (Fig. 1d) and were characterized by presence of small numbers of extravasated erythrocytes 10–20 μm from nearby cerebral vessels (Fig. 1e). BBB opening in CSD brain was further characterized by IgG deposition (Fig. 1f-i) in both parenchyma (Fig. 1h) and perivascular spaces (Fig. 1i). Spread of perivascular labeling of IgG appeared to be restricted in some cases (Fig. 1i) by vascular and astrocytic basement membranes, based on similar pattern of IgG deposition after injection into the subarachnoid space (Pizzo et al., 2018).

Microbleeds marked either by Ter119 or IgG, similar to those marked by FITC-dextran (Lehmann et al., 2018), were distributed stochastically across all anatomical regions.

### 3.3. Microarray analysis of brain endothelial cells (bEC)

**Fig. 2.**
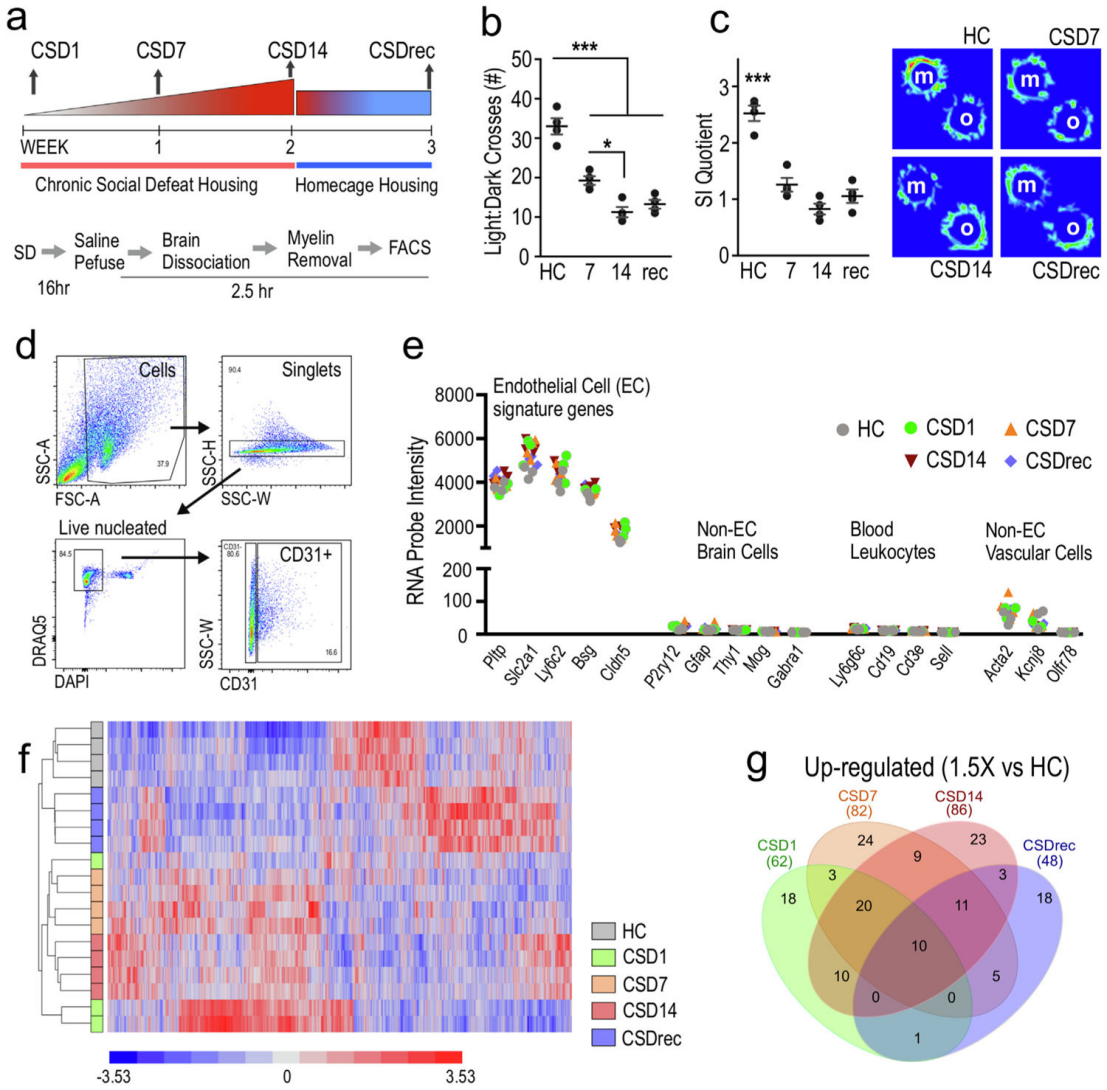
Additive exposure to CSD had distinct effects on brain endothelial cell (bEC) expression profiles. a, Experimental scheme and collection points. b,c, CSD exposed mice showed significant declines in light:dark crosses (b), and social exploration (c) compared to nonstressed mice. d, FACS gating strategy used to isolate bECs. e, Signature gene analysis of cells extracted from all groups of mice indicating that the examined populations are ECs. f, Heat maps from unsupervised clustering for all differentially expressed genes revealed treatment grouping (one-way ANOVA; false discovery rate (FDR) q < 0.05). g, Comparison of up-regulated genes with ≥ 1.5x change in gene expression compared to HC shows distinct profiles in each group. *** HC vs. all p < 0.0001, * CSD7 vs. CSD14 p < 0.05. Abbreviations: m, mouse; o, object; CSD1, 7, 14 denote number of CSD exposures in days. CSDrec are samples collected after CSD14 plus 7 days in homecage recovery.

We used microarray analysis to determine gene expression patterns in bECs isolated from mice receiving 1 (CSD1), 7 (CSD7), or 14 (CSD14) days of CSD to examine molecular events involved with microbleeds and barrier dysfunction in socially stressed mice. To gain insight in altered homeostasis and reparatory mechanism, we further examined EC expression patterns in mice receiving 7 days of HC recovery after 14 days of CSD (CSDrec). All responses were compared to HC. The experimental design is shown in Fig. 2a. CSD caused sharp declines in light:dark crosses (Fig. 2b) and social exploration (Fig. 2c) compared to HC mice at all tested time points including mice in recovery (LD crosses; one-way ANOVA F_(3,12)_ = 45.23, p = 0.0001) (SI quotient; one-way ANOVA F_(3,12)_ = 40.3, p = 0.0001). Expanded behavioral profiles of all mice and those selected for the array experiment are shown in figure S1. Fluorescence-activated cell sorting (FACS) was used to isolate bECs from enzymatically digested and myelin-removed brain (Fig. 2d, Fig. S2). Sample purity was confirmed with signature gene analysis (Fig. 2e). Gene expression profiles of purified CD31^hi^ bECs subjected to microarray were highly enriched for established bEC signature genes including *Pltp, Slc2a1, Ly6c2, Bsg,* and *Cldn5* (http://brainrnaseq.org) (Daneman et al., 2010). Expression levels of these genes were substantial in all conditions, and no significant differences in expression were observed across conditions. Signature markers for neurons, astrocytes, and oligodendrocytes were expressed at negligible levels as were markers for blood leukocyte subsets including C*d19* (B lymphocytes), *Cd3e* (T lymphocytes), *Ly6gc* (granulocytes), and *Sell* (peripheral macrophages) (Haage et al., 2019). Signature markers highly expressed in smooth muscle cells (*Acta2*, *Olfr78*) or pericytes (*Kcnj8*) (He et al., 2018; He et al., 2016) were not significantly evident in purified samples.

Analysis of purified bECs across all groups identified 3174 differentially expressed genes (One-way ANOVA; Partek, false discovery rate (FDR) q < 0.05), of which 1009 are predicted genes (Fig. 2f). Unsupervised hierarchical clustering of these transcripts showed that the biological programs governing transcript expression were different between experimental conditions and dependent on the duration of stress exposure. The dispersed CSD1 samples indicate the individual acute responses to stress that become more coordinated with subsequent, chronic exposure. Marked temporal variations in gene expression across different stress groups was further illustrated in Fig. 2g, where ~ 1/3 of highly upregulated transcripts compared to HC are unique at each time point.

**Fig. 3.**
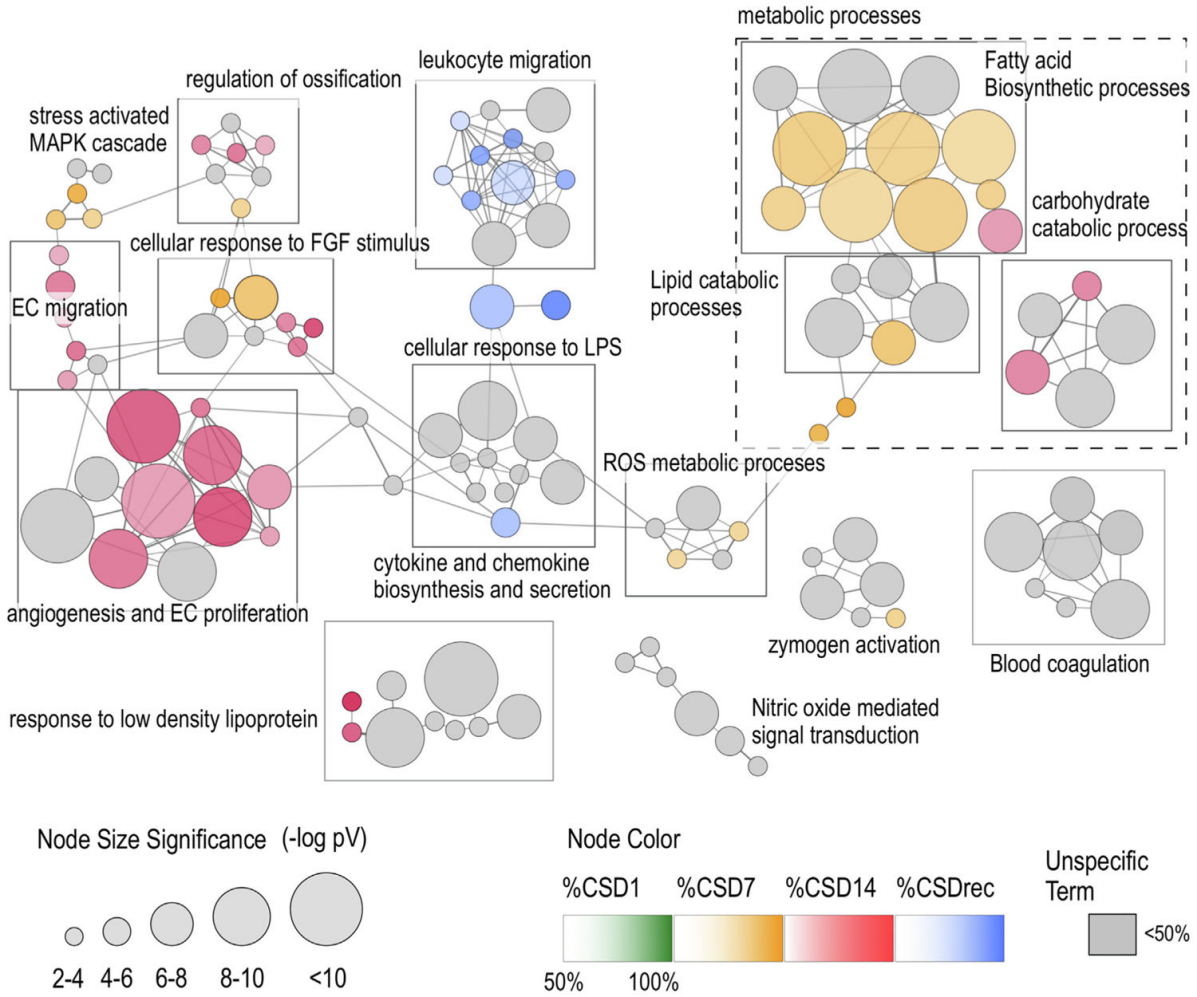
Duration of CSD exposure determines brain endothelial cell (bEC) function. Non-redundant biological processes for transcripts with ≥ 1.3 × upregulation were mapped onto a network graph for each treatment group (CSD1, CSD7, CSD14, CSDrec) using ClueGO. Nodes represent GO terms, edges represent the similarity of their associated genes, and functionally related nodes (determined by kappa score) are clustered into communities (marked with boxes where appropriate). The node size reflects the enrichment significance of the term. Node color, when evident, reflects the dominance of a treatment group for that node, at least 50% of the differentially expressed transcripts for that node need to be expressed by a single group. Gray nodes occur were no one treatment group is in the majority. Node Colors: green = CSD1, yellow = CSD7, red = CSD14, blue = CSDrec. The leading group term is based on highest significance vs. cluster. (For interpretation of the references to color in this figure legend, the reader is referred to the web version of this article.)

Next, we defined patterns of gene ontology (GO) terms that could characterize changes in brain endothelial cell (bEC) phenotype/function during the course of our stress/stress recovery paradigm using ClueGO plus CluePedia (Bindea et al., 2013; Bindea et al., 2009; Shannon et al., 2003) (Fig. 3). For this, 578 unique 1.3x upregulated transcripts were clustered by ClueGO into non-redundant enriched GO terms; roughly similar numbers of unique and overlapping transcripts were found in each treatment group (CSD1 247, CSD7 270, CSD14 290, and CSDrec 236). This analysis revealed a number of unique and shared functional biological pathways associated with each treatment. For instance, biological processes associated with metabolism were highly engaged during the CSD7 treatment cluster, angiogenic processes were dominant in the CSD14 cluster, and leukocyte migration was dominant in the CSDrec group. The network graph makes readily apparent the shared relationship between different GO communities. Substantially fewer transcripts were downregulated by stress conditions. We found 48 unique transcripts downregulated 1.3-fold or greater across all stress conditions; of these, 27 were predicted genes. No significant enriched GO terms were found in this list.

**Fig. 4.**
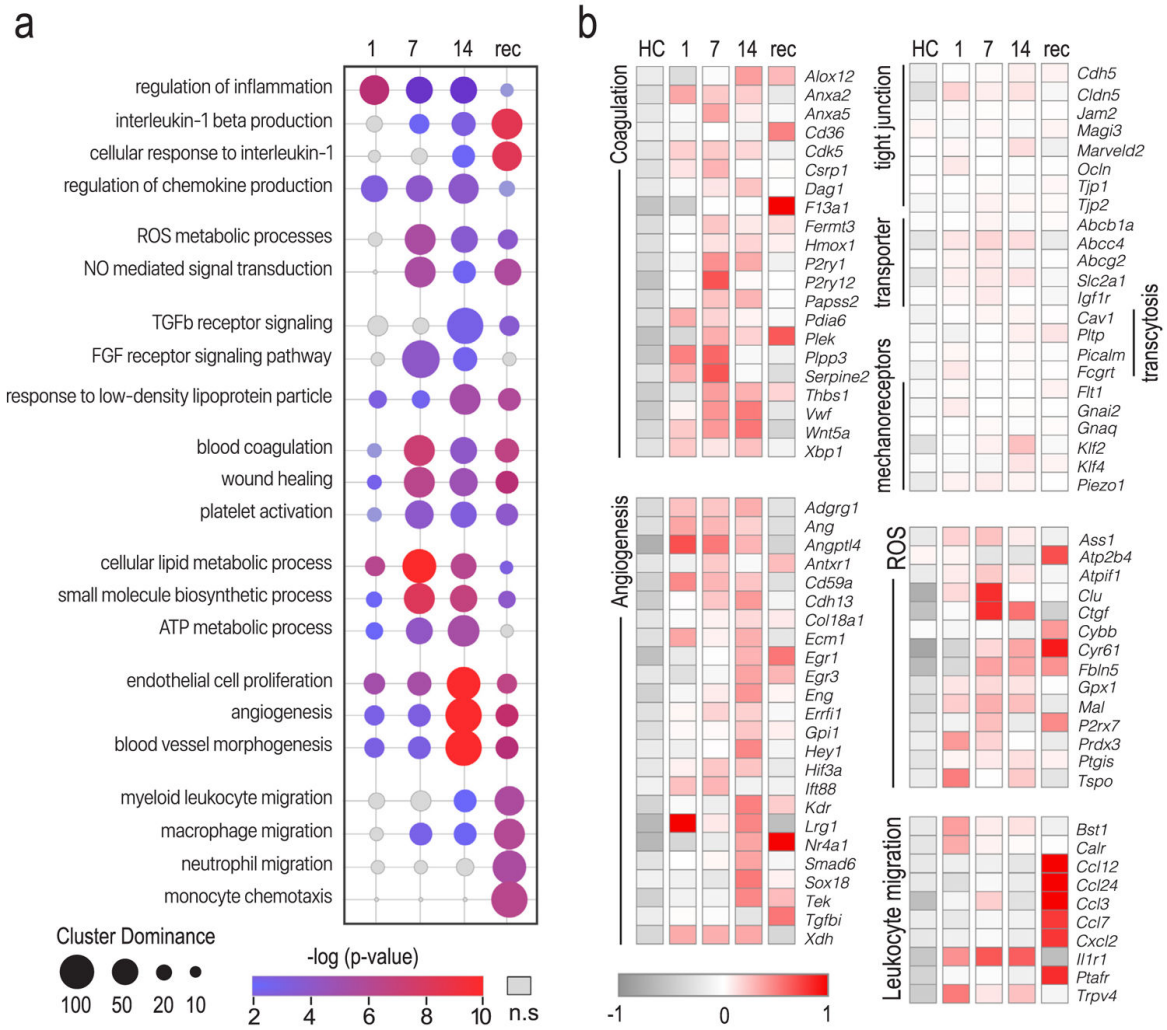
Dynamic responses of bEC reflect evolving biological processes during repeated CSD. a, Enrichment analysis for GO terms within each community was performed using ClueGO to reveal the emergence of biological processes during repeated CSD. The bubble chart shows the dominance of a GO term at each collection point in the experiment. GO terms were manually curated to represent the 7 largest communities of GO processes. Bubble size indicates the dominance of a timepoint for that term (percent of genes specific to a term expressed at that time point). Bubble color indicates p-value significance. GO enrichments are scaled by log_10_(p value) with only significant (p < 0.05) enrichments featured in color. b, Heatmap of normalized gene expression in bEC characterizing communities of GO terms identified in a. Color represents the mean fold change gene expression of a group vs. mean gene expression level of all samples. The color code (gray, low expression; red, high expression) is shown at lower left of heat map. A complete list of GO terms and associated genes is provided in Extended Fig. 4-1. Abbreviations: 1, CSD1; 7, CSD7; 14, CSD14; Rec, CSDrec. (For interpretation of the references to color in this figure legend, the reader is referred to the web version of this article.)

Terms curated from several communities of clustered GO processes shown in Fig. 3 were further examined to reveal a change in enrichment for these terms during the course of CSD. Term enrichment, determined by the number and diversity of differentially expressed genes within a term, was dynamically plotted in Fig. 4a. Two features were evident from this type of display; 1) the emergence of distinct major biological processes as mice accumulated defeat exposures, 2) different time courses for the appearance and disappearance of the biological processes over course of defeats and subsequent recovery. On day 1 of defeat exposure, functions related to inflammation were prominent. On day 7, oxidative stress (reactive oxygen species, ROS and nitric oxide, NO), metabolic processes including lipid metabolism and biosynthesis, growth factor signaling, and blood coagulation and wound healing were present. On day 14, angiogenesis was dominant.

During recovery, leukocyte migration appeared (Fig. 4a). This process is very intriguing, and we are pursuing it with a following manuscript that explores the actual leukocyte response. We hypothesize that monocytes traffic to brain after the cessation of stress to facilitate the later stages of wound healing, including removal of fibrin depositions. We further hypothesize that stress hormones released during CSD inhibit monocyte migration.

Heatmaps for individual genes that comprise the curated communities for coagulation, angiogenesis, ROS metabolism, and leukocyte migration are shown in Fig. 4b. Many transcripts belong to more than one community; their placement in the heatmap was subjective. A complete list of GO terms and associated genes is provided in Table S1. The coagulation community contains differentially expressed genes involved with hemostasis, including *Vwf* (von Willebrand factor) and the purinoceptors *P2ry1* and *P2ry12, Serpine2, Anxa2* and *Fermt3*. These transcripts have been used as biomarkers of increased risk of thrombosis in clinical settings and support the presence of clustered erythrocytes (from microbleeds) (Fig. 1). The angiogenesis community is defined by well-known angiogenic markers *Kdr* (vascular endothelial growth factor receptor 2), *Tek* (angiopoietin-1 receptor), and *Ecm1*. The leukocyte migration community is populated with numerous chemokine ligands, including *Ccl12, Ccl2, Ccl24,* and *Cxcl2.*

We further examined for expression changes in transcripts that support decline in barrier function observed in CSD mice but were not dominant in the ClueGO communities, namely genes encoding tight junctions, nutrient transporters, and transcytosis. However, no significant alteration in expression was observed between CSD and HC mice (Fig. 4b). The absence of change in tight junction proteins or transporters suggest that defects in these pathways are not responsible for stress effects on bEC function. We spent some time analyzing for changes in any structural protein, and none of our data suggested that decline in tight junction gene expression was driving the current dataset. We conclude that CSD as carried out in this study doesn’t cause expressional changes in tight junction protein transcripts like those reported in another CSD study (Menard et al., 2017), which we note was a regionally restricted analysis. Further, we strive to minimize wounding that may occur in other defeat paradigms. We have suggested that this procedural difference may underlie whether or not monocytes enter the brain (Lehmann et al., 2016). Redistribution of tight junction proteins has been reported following restraint stress (Santha et al., 2015), which might contribute to paracellular transendothelial diapedesis when it occurs (Winger et al., 2014). During recovery, the lack of change in tight junction transcription suggests that leukocyte migration into the brain parenchyma may be minimal even though endothelial chemokine transcriptional activity is high.

We identified upstream regulators for these communities using Ingenuity Pathway Analysis (IPA). An abbreviated list of predicted upstream regulators with activation z-scores ≥ 2 or ≤ −2 during at least one sampling timepoint is shown in Figure S3. Individual upstream regulators were grouped according to molecule type, and the z-scores derived from the HC vs. stress treatment groups illustrate the predicted change in regulator activity with progressive stress exposure. Upstream regulators associated with cytokines were predicted to become more prominent with increasing CSD exposure. This is notable because vessel damage or exposure to certain cytokines shift endothelial cells towards a procoagulant prothrombotic phenotype (Michiels, 2003). Several predicted factors associated with platelet aggregation (PAF), coagulation (F2R), and fibrinolysis pathways (PDGF BB, TP53) further support thrombosis as a key driver for CSD transcriptional profile. Notably, the z-scores for many but not all of these factors trended higher with increasing CSD exposure and declined in recovery samples. Other factors (PPARGC1, AGT) and drugs (losartan) related to vasoconstriction (Lu et al., 2016; Oberkofler et al., 2003) suggest that alterations in blood pressure, such as those seen in hypertension, might underlie CSD effects on bECs. The opposing antihypertensive action of losartan is reflected by the negative z-score. Other factors linked to angiogenesis (FOXO1, and TP73) were predicted to become more active during CSD14, supporting the enrichment analysis in Figs. 3 and 4.

**Fig. 5.**
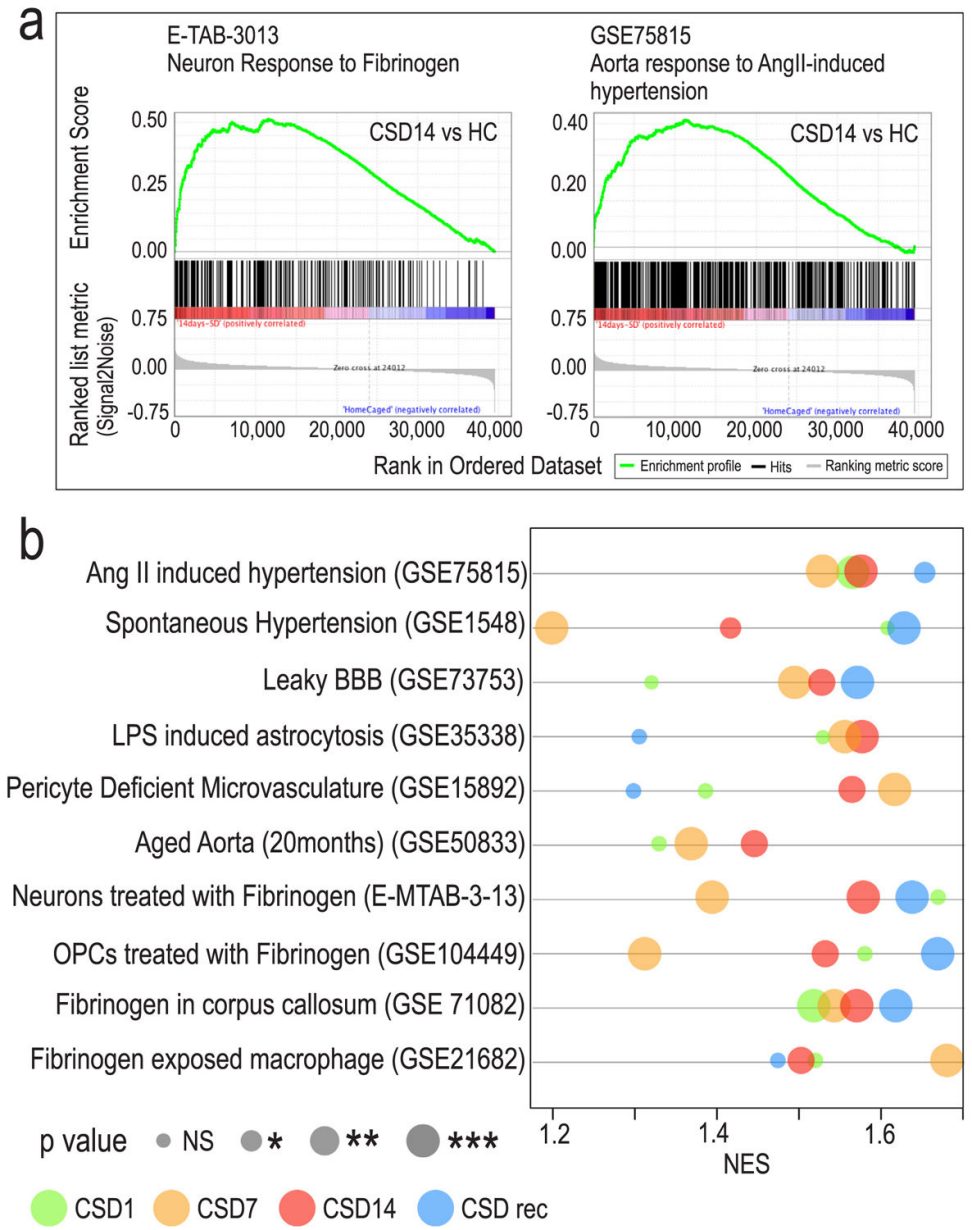
CSD produces a bEC phenotype seen in hypertension, neuroinflammation, and BBB disruption disease models. Microarray data were analyzed using gene set enrichment analysis (GSEA) software to identify functionally related gene sets with statistically significant enrichment in stressed bEC compared with several models of vascular pathology. a, GSEA plots show bEC transcriptional responses to CSD14 are robustly concordant to neurons exposed to fibrinogen or vascular tissues in Angiotensin II-dependent hypertension. b, The normalized enrichment score (NES) represents the degree to which a specific set is represented at the extremes (high or low) of the ranked gene list. Bubble plot shows that gene sets from CSD exposed bECs highly correlate with gene sets from several models of hypertension, leaky BBB, vascular aging, and cellular responses to fibrinogen exposure. Bubble color indicates sampling point: green, CSD1; yellow, CSD7; red, CSD14; blue, CSDrec. Bubble size indicates p value: ns not significant, * < 0.05, ** < 0.01, *** < 0.001. (For interpretation of the references to color in this figure legend, the reader is referred to the web version of this article.)

We used GSEA to reveal published expression profiles with shared similarities to our dataset and characterize bEC phenotypes produced by CSD exposure. The transcriptome of published experimental models for thrombosis, hypertension, and BBB dysfunction were remarkably similar to the transcriptional profile produced by CSD (Fig. 5a,b). At the disease level, genes upregulated by CSD were similar to genes upregulated in cerebral microcapillaries of stroke-prone spontaneously hypertensive rats (GSE1548) and in the arteries of hypertensive mice infused with angiotensin II (GSE75815). The CSD transcriptome profile was also concordant in mouse mutant models with increased BBB permeability (GSE73753 and GSE15892). A key feature of BBB disruption is the influx of blood proteins into brain, including the coagulation factor fibrinogen. Fibrinogen-induced transcriptome changes in numerous cell subtypes (Fig. 5b) was strikingly similar to CSD-induced changes in bECs, highlighting this coagulation factor as a key player in our dataset.

### 3.4. Immunohistochemistry shows changes in angiogenesis and fibrinogen accumulation.

**Fig. 6.**
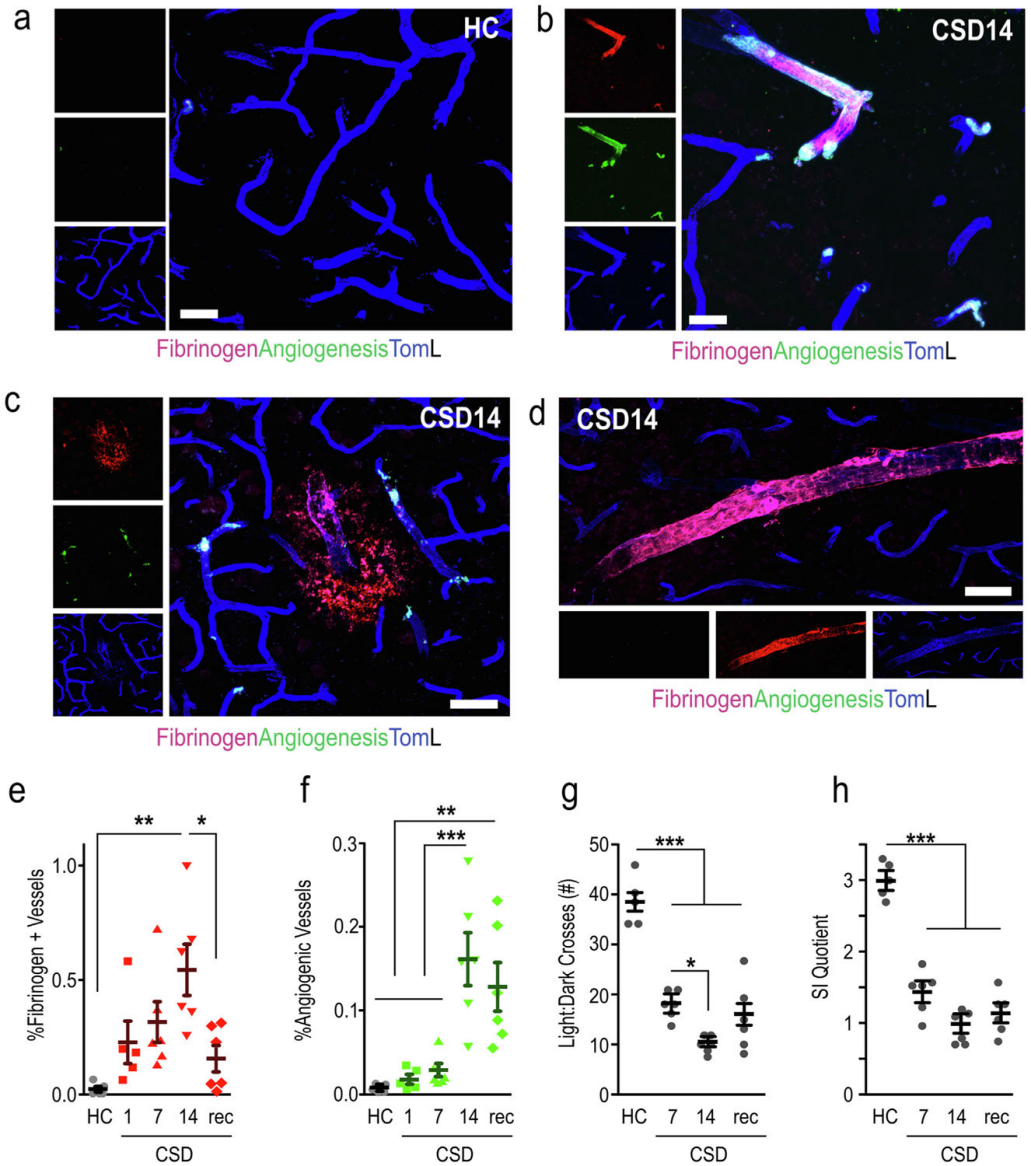
CSD causes brain vascular fibrinogen deposition and angiogenesis in a dose-dependent manner. a, Minimal detection of fibrinogen and angiogenesis markers (CD202, CD105, and VEGFR2) detected in brain vasculature of nonstressed mice. b, Perivascular fibrinogen deposition and angiogenesis in CSD exposed mouse. c, Example of a parenchymal fibrinogen leak surrounded by angiogenesis markers in a CSD brain. d, Perivascular fibrinogen accumulation with minimal angiogenesis in CSD exposed brain. e, CSD exposure causes significant fibrinogen deposits, and this occurs in a dose-dependent manner. f, Brain vascular angiogenesis increases with increasing CSD exposure. g,h, CSD-exposed mice showed significant declines in light:dark crosses (g), and social exploration (h) compared to nonstressed mice. Scale bar = 20 μm for (a,b) and 40 μm for (d,f). N = 5 for HC and CSD1, N = 6 for all other groups. One-way ANOVA: * p < 0.05, ** p < 0.01, *** p < 0.001.

Fibrinogen deposition is a key process of blood coagulation, stabilizing the hemostatic plug and providing a matrix for wound and vessel repair. Our bioinformatic analyses highlighted coagulation events and angiogenesis as key consequences of CSD, and we evaluated these events in a fresh cohort of stressed and recovered mice. Fibrinogen deposition was nearly nonexistent in non-stressed samples (Fig. 6a). In stressed brains, perivascular (Fig. 6b,d) and parenchymal depositions (Fig. 6c) were observed. Deposition significantly increased with accumulating CSD exposure, and it rapidly declined in stress recovery brains; F_(4,23)_ = 11.1, p = 0.0001 (Fig. 6e). Deposition was stochastically distributed in a pattern closely resembling that of the erythrocyte clusters and IgG deposition (Fig. 1).

To examine evidence for angiogenesis in the same sections, three antibodies were used in a cocktail—CD202b (Tie2), CD105 (Endoglin), and VEGFR2. All three respective genes (*Tek, Eng,* and *Kdr*) were upregulated at day 14 of CSD (Fig. 4b), and all are found in the ClueGO cluster “positive regulation of vasculature development.” In brain, the genes are selectively expressed in endothelial cells (http://www.brainrnaseq.org). Immunostaining showed that the trend for angiogenesis was similar to the accumulation of fibrinogen deposits, but it remained elevated during recovery; F_(4,23)_ = 5.56, p = 0.0028 (Fig. 6f). Both fibrinogen and angiogenesis were substantially colocalized (Fig. 6 b), but not exclusively (Fig. 6d), suggesting extravasated fibrinogen may guide neo-revascularization. Behavioral analysis of this cohort showed that all CSD exposed groups had sharp declines in LD crosses (Fig. 6g) and SI scores (Fig. 6h) compared to HC mice (LD crosses; one-way ANOVA F_(3,19)_ = 43.1, p = 0.0001) (SI quotient; one-way ANOVA F_(3,19)_ = 52.2, p = 0.0001).

A simple linear regression was carried out to investigate the relationship between immunohistochemical stains (fibrinogen and angiogenesis) and behavioral measures (SI and LD crosses) in cohorts exposed to CSD. The scatterplots (Fig. S4) revealed a strong negative linear relationship between fibrinogen and social interaction [F_(1,16)_ = 4.55, p = 0.048, R^2^ = 0.22, which was confirmed with a Pearson’s correlation coefficient r of −0.469 (p = 0.001) (Fig. S4a). That relationship also held between fibrinogen and LD crosses [F(1,16) = 4.35p = 0.05, R^2^ = 0.21 (Fig. S4b). A strong negative relationship was observed between angiogenesis and social interaction [F(1,16) = 17.93, p = 0.0006, R^2^ = 0.53), confirmed by Pearson’s r of − 0.73, (p < 0.0001)(Fig. S4c). Linear regression between angiogenesis and LD crosses was close to significant [F (_1,16_) = 4.15, p = 0.054, R^2^ = 0.22 (Fig. S4d). The inverse relationship was confirmed by Pearson’s r (r = −0.56, p = 0.013; r = −0.47, p = 0.05) for LD crosses versus angiogenesis and fibrinogen, respectively. The data show that severity of psychological effect as measured behaviorally correlates with severity of microbleeds. These results show that SI scores could be a predictor of fibrinogen deposition and in turn suggest the degree of fibrinogen deposition accrued during CSD could predict behavior.

## 4. Discussion

Pathological disruption of the BBB is a feature of many neurological disorders (de Vries et al., 2012), and it may also occur in psychiatric disorders (Kealy et al., 2020). Using CSD, a well-established mouse psychosocial stress paradigm that confers depressive-like, anxiety-like, and asocial behavioral features seen in depression (Golden et al., 2011; Schloesser et al., 2010), we showed that chronic defeat causes microhemorrhages and leakage of plasma immunoglobulins across the BBB into perivascular and parenchymal spaces. We tracked changes in the transcriptional profile of bECs isolated from mice exposed to varying durations of CSD and during recovery following CSD. This strategy revealed how stress impacts brain vasculature over time. Bioinformatic analysis of the bEC transcriptome showed a temporally orchestrated sequence of biological pathways directing the vascular response to injury. This is the first methodological examination of changes in brain vascular programming during psychosocial stress and stress recovery.

Analysis of genes differentially expressed by bECs during CSD revealed pathways of inflammation, oxidative stress, growth factor signaling, and wound healing, which includes platelet aggregation, hemostasis, fibrinogen deposition, and angiogenesis. Histochemical analysis of brains from stressed mice showed patterns of widespread, scattered BBB leakage and parenchymal erythrocyte clustering that were paralleled by immunohistochemical staining of fibrinogen deposition and of angiogenesis markers. These observations support the hypothesis that the brain’s immune response to the adverse effects of psychological stress, evinced by gene profiling of microglia from CSD-S mice (Lehmann et al., 2018), is driven in part at least by vascular damage and blood leakage across the BBB.

GO analysis of bEC expression patterns indicated that the processes of platelet activation and blood coagulation appear early in CSD. When endothelial damage occurs, platelets come into contact with exposed collagen and von Willebrand factor, become activated, clump, and initiate a blood clot. The pathogenesis of bleeding and thrombosis is driven by the interplay of blood composition, vessel wall components, and blood flow (Wolberg et al., 2012). Abnormalities in each of these factors have been reported in stressed rodents (Finnell et al., 2017; Giannarelli et al., 2017; Heidt et al., 2014; Niraula et al., 2018).

Initiation of angiogenesis quickly follows vascular injury. Normally, quiescent endothelial cells transition to an activated phenotype and elevate their metabolism to meet the energetic and biosynthetic needs (Falkenberg et al., 2019). This was shown by numerous metabolic process that emerged at CSD7, persisted at CSD14, and returned to quiescence during recovery (Figs. 3, 4). Angiogenesis is a critical component of wound healing, and numerous GO pathways defining this process including EC proliferation were dominant at CSD14. Fibrin deposited during the early wounding periods forms a provisional matrix for migrating and sprouting endothelial cells during tissue repair (Hadjipanayi et al., 2015; Tonnesen et al., 2000). The overlap of fibrin deposition and angiogenesis markers (CD202, CD105, and VEGFR2) was evident histologically (Fig. 6).

During recovery after stress cessation, behavioral deficits were still present (Fig. 2b,c) while angiogenesis, inflammation, and ROS activity persisted (Figs. 4 and 6E). Subsiding expression of metabolic- and coagulation-linked transcripts suggest that vascular damage had declined. Peripheral leukocyte activation became evident in recovery. Peripheral macrophages may assist in vascular repair (Liu et al., 2016; Russo et al., 2018) and fibrin endocytosis (Motley et al., 2016).

The stress-induced bEC transcriptional profiles were recapitulated in datasets mined from hypertension, vascular inflammation, and aging (Fig. 6), implying that disruptions in blood flow, hemodynamic events, and inflammation drive the BBB breakdown in the CSD model. If such peripheral events drive the vascular pathology, that would “explain” the stochastic distribution of microbleeds (Fig. 1). Alternatively, centrally originating influences might be expected to produce a regionally restricted pattern of vascular alterations. Two recent studies reported stress-induced vascular changes localized to brain regions associated with emotional processing (Menard et al., 2017; Pearson-Leary et al., 2017). Other studies of stress-induced CNS inflammation often find regionally restricted microglial activation, but there is limited agreement about its anatomical landscape (Walker et al., 2013).

The lack of agreement among these studies and the observed widespread distribution of microbleeds suggests there is a significant peripheral component to the endothelial response. Perhaps stress-induced elevated activities of the hypothalamic-pituitary axis and sympathetic nervous system, which drive endocrine, immune, and cardiac fluctuations, can precipitate hypertension and endothelial dysfunction (Brooks et al., 2018; Burrage et al., 2018). Social stress in a rat defeat model caused sympathomimetic changes in mean arterial pressure and heart rate (Finnell et al., 2017). CSD also elevated levels of circulating proinflammatory cytokines (Brachman et al., 2015; Hodes et al., 2014) and glucocorticoids (Lehmann et al., 2013b). Cytokines (Lopez-Ramirez et al., 2013) and glucocorticoids (Salvador et al., 2014) can directly impact bECs, and glucocorticoid feedback to the brain can alter the central inflammatory state (Frank et al., 2019; Frank et al., 2012).

In humans, brain microhemorrhages are seen by magnetic resonance imaging (MRI) (Haller et al., 2018) and in post-mortem histological tissue (Cullen et al., 2005; van Veluw et al., 2016). These and white matter hyperintensities are seen in depression, especially in the elderly (Herrmann et al., 2008; Thomas et al., 2002), though they exist also in younger clinical populations (Wang et al., 2019). Microbleeds characterize atherosclerosis (Conijn et al., 2012) and small vessel disease (SVD) (Thrippleton et al., 2019). Biomarkers used to identify SVD are prevalent in our model of CSD including microglial CD68 upregulation (Fernando et al., 2006; Lehmann et al., 2016), circulating vWF (Lavallee et al., 2013), and extravascular plasma proteins fibrinogen and IgGs (Tomimoto et al., 1996; Utter et al., 2008).

CSD-induced microbleeds, being little more than erythrocyte clusters and circumscribed IgG deposition, are smaller than those characterized in vascular disease. CSD-induced bleeds more closely resemble experimental laser-induced microbleeds (Ahn et al., 2018) or mild meningeal vascular injuries (Russo et al., 2018), which are discrete and resolved within two weeks. We surmise that the CSD-induced bleeds are being actively resolved, in part accounting for the small number detected at any particular time point and the variations in their appearance. Studies of longer CSD durations may indicate whether vascular damage takes on a more permanent character.

The data leave many unanswered questions suggesting future experiments. The relationship between fibrinogen deposition in brain vasculature and declines in mood suggests that fibrinogen entry provides an inflammatory signal that could transmit effects of CSD across the BBB. Fibrinogen in other disease states causes neuroinflammation, apparent as microglial activation (Merlini et al., 2019; Petersen et al., 2018). Elevated plasma fibrinogen is associated with severity of psychological distress and depression (Wium-Andersen et al., 2013). Fibrinogen leaks observed in the current experiment may be responsible for the microglial transcriptional profile and behavior observed in CSD-S mice in previous studies (Lehmann et al., 2018). Future work will explore the effects of fibrinogen on the brain environment and behavior and will focus also on the contributions of other cells of the neurovascular unit (NVU), which, in addition to endothelia, comprises smooth muscle cells, pericytes, astrocytic end feet, and basement membranes (Blanchette and Daneman, 2015). Finally, effects of manipulations such as environmental enrichment and blood pressure control will be assessed as therapeutic treatments.

## Supplementary Material

1
