# Supplementary material for: Analysis of cerebrovascular dysfunction caused by chronic social defeat in mice
Source: Brain Behav Immun. Author manuscript; Available in PMC 2020 Aug 10. (PMC7416466; doi:10.1016/j.bbi.2020.05.030)
Supplement: 1 [file NIHMS1595434-supplement-1.pdf]

Supplemental Figures for M.L. Lehmann et al.
Analysis of cerebrovascular dysfunction caused by chronic social defeat in mice

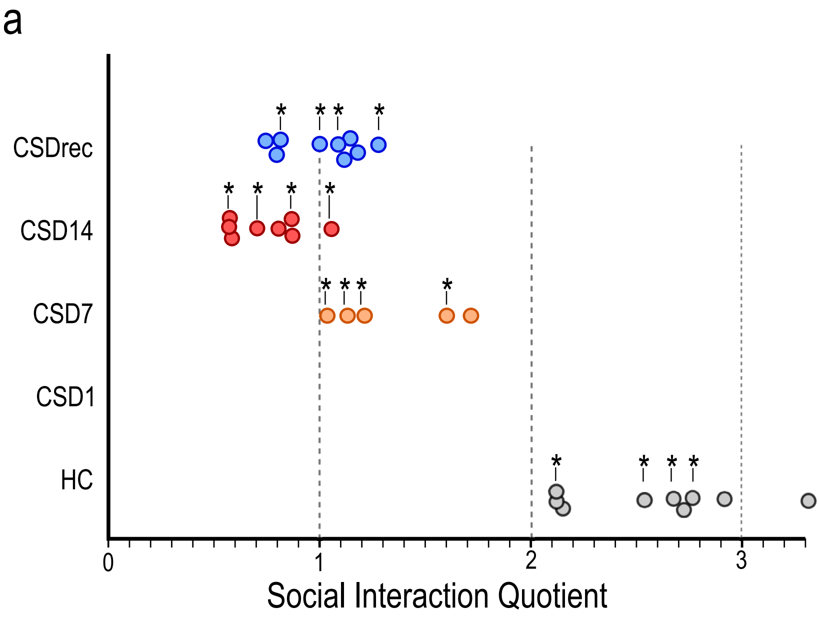

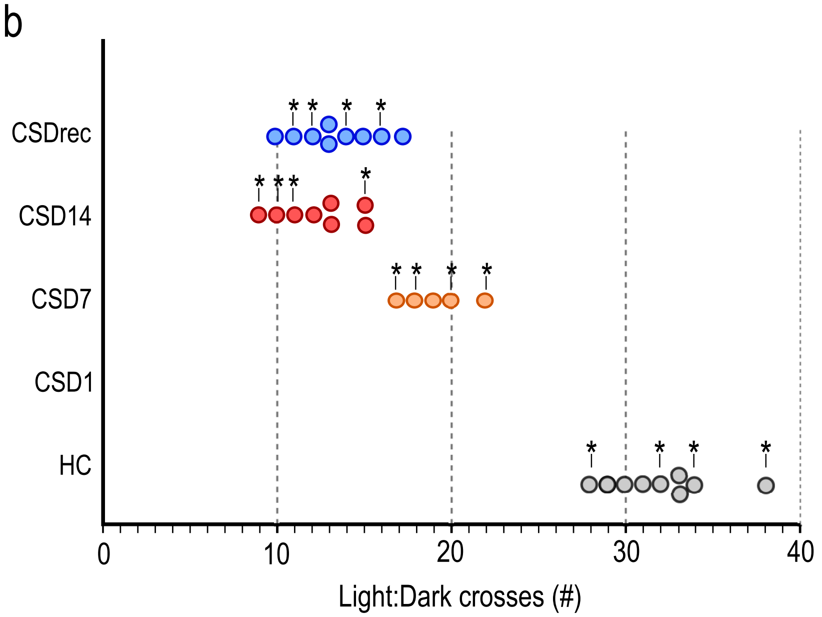

Figure S1. Distribution of behavior in mice phenotyped for the microarray experiment. a, Scatter dot plot represents the distribution of social interaction quotients (SI) from individual mice across all groups. b, scatter dot plot showing the distirbution of crosses between light and dark compartments in the light:dark test from individual mice across all groups. (*) signifies the behavior of animals chosen for the microarray experiment. As described in the methods section, all mice except groups receiving a single defeat were phenotyped the day prior to bEC collection. Gray, HC; orange, CSD7; red, CSD14; blue, CSDrec..

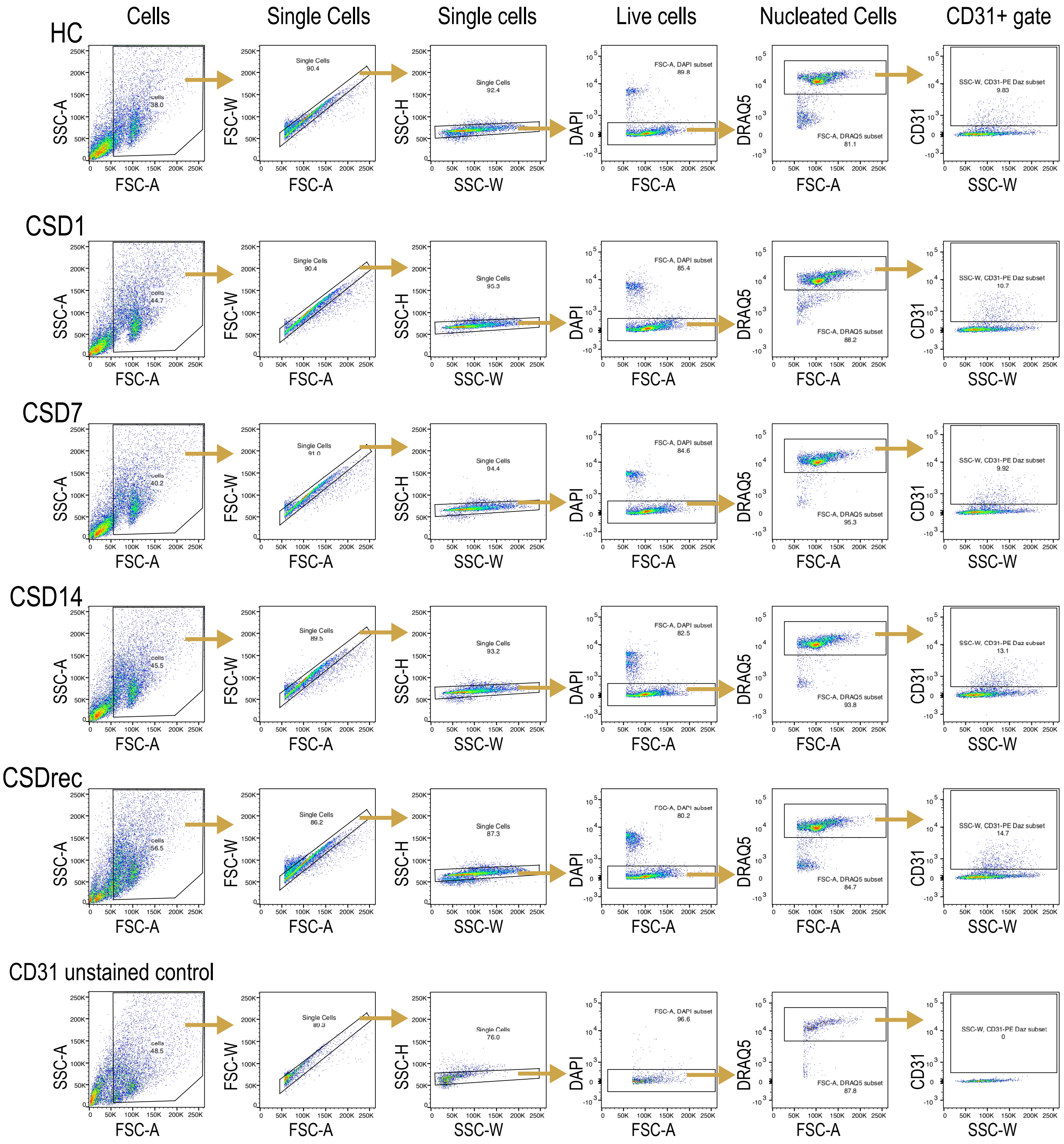

Figure S2. Gating strategy for isolating viable brain endothelial cells (bECs) with fluorescence-activated cell sorting. Representative bivariate plots detailing flow selection are shown for each experimental group.

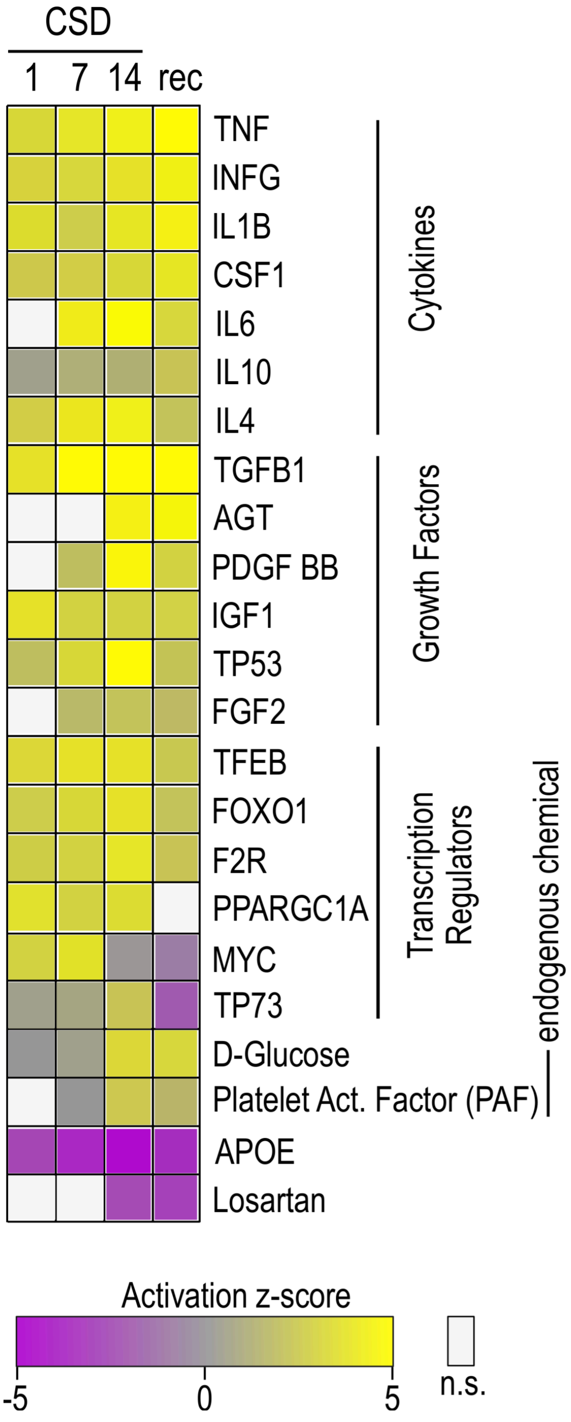

Figure S3. Temporal changes in predicted upstream regulators for bEC transcriptional changes during progressive CSD exposure. The abbreviated list of upstream regulators with a $p < 0.05$ and an absolute $z \geq 2$ or ≤ -2 in at least one sampled timepoint after 1, 7, 14 CSD or 14CSD + 7 days of recovery as compared with nonstressed bECs. Rows represent individual regulators, and columns represent time interval sampled. Colors indicate higher or lower z-scores. Negative z-scores reflect regulators that would induce transcriptional changes opposite to CSD. Regulators are grouped by molecule type.

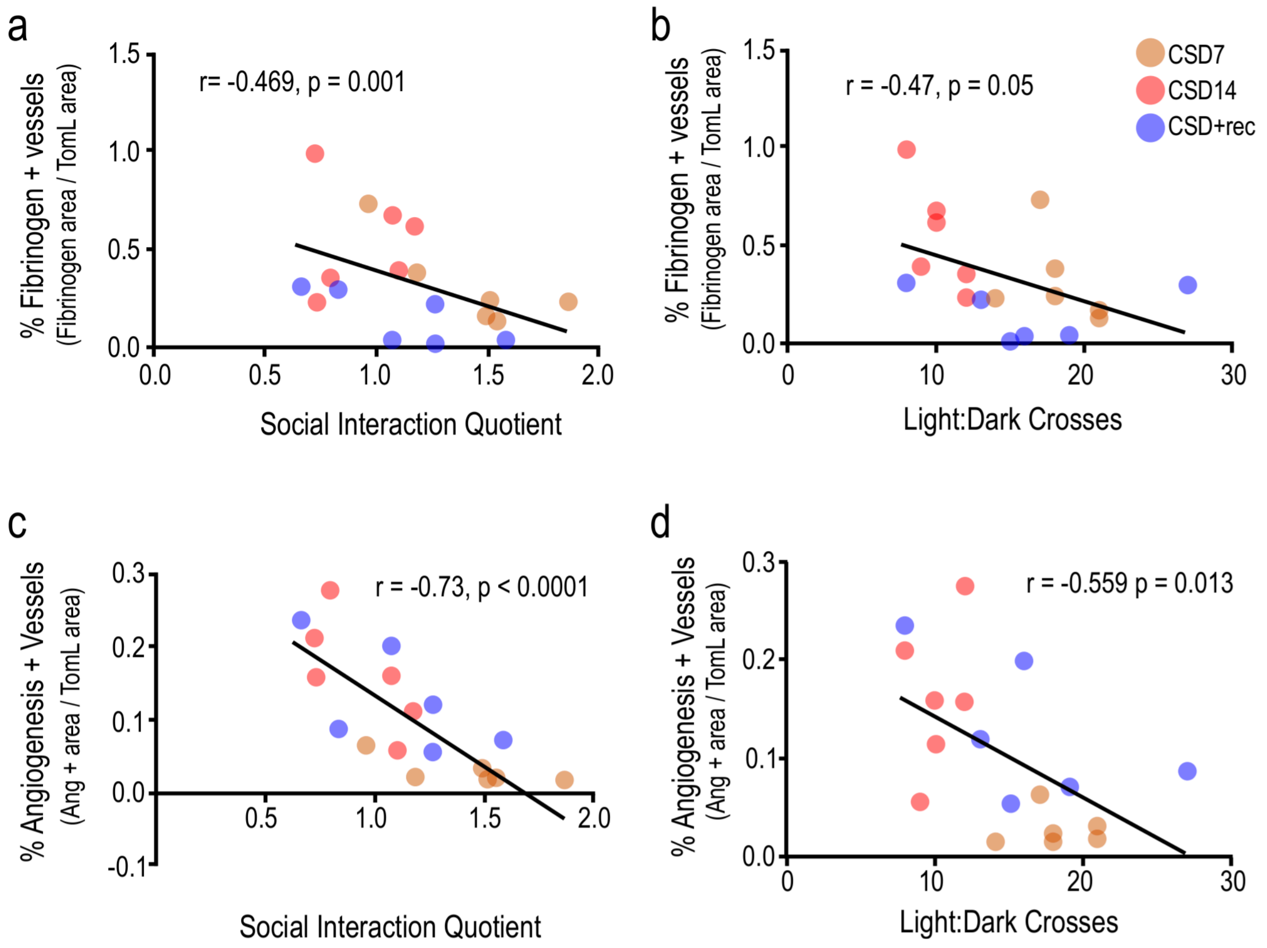

Figure S4. Linear regression analysis revealed a significant inverse relationship between vascular deposition of fibrinogen in CSD-exposed mice and behavioral performance in the social interaction (SI) test and light:dark (LD) test (a, b). A significant inverse relationship between the number of angiogenic-positive vessels and social interaction performance (c) and light:dark crosses (d) was also observed. r = Pearson's correlation coefficient.
